# Explainable correlation-based anomaly detection for Industrial Control Systems

**DOI:** 10.3389/frai.2024.1508821

**Published:** 2025-02-04

**Authors:** Ermiyas Birihanu, Imre Lendák

**Affiliations:** Data Science and Engineering Department, Faculty of Informatics, Eotvos Lorand University, Budapest, Hungary

**Keywords:** anomaly detection, correlation, explainable, Industrial Control System, root cause analysis

## Abstract

Anomaly detection is vital for enhancing the safety of Industrial Control Systems (ICS). However, the complicated structure of ICS creates complex temporal correlations among devices with many parameters. Current methods often ignore these correlations and poorly select parameters, missing valuable insights. Additionally, they lack interpretability, operating efficiently with limited resources, and root cause identification. This study proposes an explainable correlation-based anomaly detection method for ICS. The optimal window size of the data is determined using Long Short-Term Memory Networks—Autoencoder (LSTM-AE) and the correlation parameter set is extracted using the Pearson correlation. A Latent Correlation Matrix (LCM) is created from the correlation parameter set and a Latent Correlation Vector (LCV) is derived from LCM. Based on the LCV, the method utilizes a Multivariate Gaussian Distribution (MGD) to identify anomalies. This is achieved through an anomaly detection module that incorporates a threshold mechanism, utilizing alpha and epsilon values. The proposed method utilizes a novel set of input features extracted using the Shapley Additive explanation (SHAP) framework to train and evaluate the MGD model. The method is evaluated on the Secure Water Treatment (SWaT), Hardware-in-the-loop-based augmented ICS security (HIL-HAI), and Internet of Things Modbus dataset using precision, recall, and F-1 score metrics. Additionally, SHAP is used to gain insights into the anomalies and identify their root causes. Comparative experiments demonstrate the method's effectiveness, achieving a better 0.96% precision and 0.84% F1-score. This enhanced performance aids ICS engineers and decision-makers in identifying the root causes of anomalies. Our code is publicly available at a GitHub repository: https://github.com/Ermiyas21/Explainable-correlation-AD.

## 1 Introduction

In complex industrial systems, anomalies are not isolated incidents and pose significant challenges due to their interconnected nature, device dependencies, and diverse data streams (Guo et al., 2018; Birihanu et al., 2022). Anomalies can spread from one sensor or actuator to others, causing increasingly severe problems within devices. Efficient algorithms are needed to handle diverse data types, including continuous and discrete variables. Capturing correlations between devices in multivariate time series becomes crucial. However, dynamically discovering, representing, and detecting these correlations is a complex task due to varying device functionalities and dynamic relationships. Consequently, correlation-based anomaly detection emerges as a powerful tool to identify anomalous correlation patterns (Guo et al., 2020).

When dealing with anomaly detection scenarios in complex infrastructure systems, there are three primary issues to consider. Firstly, obtaining labeled datasets can be challenging. Secondly, the devices within the system do not function independently but are interconnected. Lastly, the data collected from each device may vary in properties, such as being static or time series, and can also be of different types, including continuous or discrete (Qin et al., 2022). In industrial control systems (ICS), some equipment parameters show a hidden correlation that remains stable during normal operations but changes significantly during abnormal states. This hidden correlation refers to relationships between latent variables in a statistical model, not directly observable but inferred from connections between observed variables influenced by the underlying latent variables (Zhong et al., 2016).

Detecting abnormal conditions in the ICS through individual sensor/actuator parameters is possible, but some abnormalities require the operators long-term experience such as in-depth knowledge of industrial processes, historical context, emergency response protocols, and alarm interpretation. To handle the complexity of abnormal ICS operations, internal parameter correlations are explored. Detecting abnormalities during steady-state operations has received significant research attention, as stealthy attacks (Raman et al., 2020) that can evade detection for extended periods can occur, however identifying abnormalities during dynamic changing conditions poses challenges. The correlation coefficient method is commonly used to investigate the internal relationships between ICS parameters and represent correlations between variables. The most anomaly detection methods for ICS focus on all features (Elnour et al., 2020; Perales Gómez et al., 2020) or stage-wise features (Bernieri et al., 2019; Raman et al., 2020; MR et al., 2020) separately within the given dataset and only focus on individual devices, not how devices correlate or how attacks take place over time (Jadidi et al., 2023). However, for precise anomaly detection, there is a need to develop a method that can analyze a large volume of sensor information and handle complex nonlinear coupling systems effectively (Zheng et al., 2020).

Authors in Zhao et al. (2017) mentioned anomaly detection can be approached using two methods: statistical and machine learning. Existing anomaly detection methods for ICS are not scalable or flexible, and they mainly focus on detecting point or collective anomalies (Wang et al., 2023). Collective anomaly detection is more challenging because it requires the exploration of the data structure and design of an unlikeliness measure for observations. Correlated anomaly detection is a type of group anomaly detection that measures unlikeliness solely based on correlation (Chen et al., 2018). Our focus is on correlation analysis of ICS data for analyzing complex equipment due to three key reasons. Firstly, machine sensors and/or actuators are typically correlated, reflecting the machines mechanisms and operating conditions. Secondly, correlations undergo abrupt changes during anomalies, making them highly sensitive to equipment status variations. Therefore, incorporating explainable into correlation anomaly detection enhances the trustworthiness and usability of the model. The ability to explain causal relationships within the data is a key aspect of this approach. Lastly, correlation analysis is simple to implement, offering a cost-effective and real-time solution.

High data dimensionality poses challenges, as models developed using supervised and/or unsupervised methods are often considered “black boxes,” making it difficult for humans to interpret the results and findings. Explainable Artificial Intelligence (XAI) offers a solution to this problem by helping us understand and explain how algorithms work (Hoang et al., 2022). The concept and implementation of explainable anomaly detection have been studied for deep learning approaches (Hoang et al., 2022; Khan et al., 2021), but not for correlated-based anomaly detection on ICS. Stages in an ICS include various equipment and will vary depending on the industry, application, and complexity of the system. The sensors and actuators in an ICS are correlated with each other across the current stage and beyond, extending into subsequent stages. Sensor and actuator features can change individually or together, reflecting the physical changes in the ICS state. The proposed solution in this study can represent these high-level relationships between features using correlation values that correspond to the normal state of the ICS. During an attack, if ICS features are modified by an attacker, some of the measured correlation values may deviate from those of the normal state, indicating an anomaly. In addition to the challenges of optimizing the probabilistic model and determining the optimal window size for data partitioning, which are crucial for effective anomaly detection, the root cause features for anomalies in ICS have not been thoroughly investigated. This lack of explain-ability hinders real-time deployment, user understanding, and the ability to make informed decisions regarding anomalies. As a result, Explainable anomaly detection (XAD) is gaining significant attention as researchers integrate such approaches into ML models.

Enhancing the effectiveness and efficiency of machine learning models involves optimizing computational time, memory, and energy utilization, particularly when dealing with the challenge of training on extensive datasets over prolonged periods using sliding windows. This optimization not only helps to prevent diminishing model complexity and overfitting but also ensures efficient resource allocation, a critical consideration for small ICS devices deployed across various applications due to their constrained computational resources and memory capacity (Takele and Villány, 2023).

The current anomaly detection studies have not addressed issues such as identifying the complex correlation relationships within ICS devices, pinpointing the root cause of anomalies, and operating efficiently with limited resources. This study addresses these limitations by proposing an approach for enhancing the performance of anomaly detection models in ICS. In addition, an interpretable correlation method that effectively addresses these challenges is presented and is applicable in ICS. Furthermore, the root causes of anomalies are investigated by identifying causal features. The study also considers the memory usage and execution time of the proposed method across sliding windows, ensuring its practical applicability in resource-constrained environments. In this study the optimal window size of the data is determined using Long Short-Term Memory Networks—Autoencoder (LSTM-AE) and the correlation parameter set is extracted using the Pearson correlation. A Latent Correlation Matrix (LCM) is created from the correlation parameter set and a Latent Correlation Vector (LCV) is derived from LCM. Based on the LCV, a multivariate gaussian distribution (MGD) is developed and anomalies are identified using an anomaly detection module that incorporates a threshold mechanism using alpha and epsilon values. The MGD model is trained and evaluated using a novel set of input features extracted using the Shapley Additive explanation (SHAP) framework.

The contributions of this study are as follows:

- The study proposes an anomaly detection method that integrates LSTM-AE and multivariate Gaussian distribution (MGD) methods to enable timely intervention and minimize disruptions in ICS.
- The proposed method employs a novel variable correlation analysis approach, which enable the detection of complex anomalies, including hidden anomalies, in ICS systems.
- Feature importance analysis with XAD can identify the key drivers of anomalies in the data, which empowers us to make more informed and effective decisions.
- The study evaluates the proposed method's memory usage and execution time across sliding windows, ensuring its feasibility in resource-constrained environments.

## 2 Literature review

### 2.1 Anomaly detection approaches

Anomaly detection approaches can be categorized into three types: knowledge-based, model-based, and data-driven approaches (Zhong et al., 2021). While most researchers utilize a data-driven training approach for anomaly detection, correlated anomalies have received less attention. Correlation measures the similarity and relationships between two variables or time series. Positive correlation indicates that both time series evolve in the same direction, whereas negative correlation implies that they evolve in opposite directions. Recent studies on correlation-based anomaly detection for flight data have explored correlation coefficients (Zhong et al., 2016) and work cycles (Ding et al., 2014) for anomaly identification.

Correlated anomaly detection (CAD) in data streams assumes that normal data entries in data streams are weakly correlated or not strongly correlated most of the time, considering strong correlations as unlikely and anomalous. A group of data entries is classified as correlated anomalies if they exhibit strong internal correlations (Chen et al., 2018). In Hanni (2020), the author proposes a correlation anomaly detection approach using six classical machine learning algorithms: histogram, Gaussian mixture, one-class support vector machine, isolation forest, and Robust PCA. The focus of the researchers is to detect significant deviations of data from the normal distribution in the context of multiple time series. Instead of solely exploring isolated time series for point outliers or anomaly patterns, their focus lies on capturing anomalies that exhibit a significant deviation from the expected behavior between multiple time series. Multivariate anomaly detection can be done by converting the data to a univariate vector and then applying a conventional univariate anomaly detection method (Ding et al., 2019).

Study Li et al. (2022) proposes the analysis of correlation characteristics within a multi-sensor system to identify and detect anomalies. This approach aims to leverage the correlation patterns observed across multiple sensors, as they provide valuable insights into the propagation of anomalies or faults within the system. By examining these correlation characteristics, the researchers anticipate uncovering important clues that can aid in identifying and understanding anomaly propagation. The authors Ding et al. (2014) and Zhong et al. (2016) employed latent correlation anomaly detection to discover correlations between variables. The method involves calculating correlation coefficients (CC) between all sensor pairs and transforming them into a latent correlation vector. Subsequently, a probabilistic model is applied to the vector for anomaly detection. Evaluation using a real flight dataset demonstrated improved detection accuracy for this approach. However, they manually determined the window size and used simulated data for their analysis.

In statistics, a normal or Gaussian distribution is a continuous probability distribution for a real-valued random variable (Koh, 2014). MGD is a statistical concept used to describe data patterns in multiple dimensions (Tong and Tong, 1990). It extends the idea of the normal distribution to higher dimensions, where data points cluster around a mean value. These variables are interrelated, so changes in one can impact others. It is defined by the mean vector and variance-covariance matrix, representing average values and relationships between variables. This distribution is widely used in data analysis and modeling with correlated variables.

Previous studies have explored anomaly detection using correlation-based approaches on various datasets. However, additional studies are required to adapt existing anomaly detection methods for ICS data. For ICS devices, identifying the correlation between features and identifying the root cause of anomalies are both essential for maintaining system health, while resource optimization is critical. This study aims to fill this gap by focusing on correlation-based anomaly detection in multidimensional time series data from ICS environments, while also considering a resource-efficient approach.

### 2.2 Explainable anomaly detection

Explainable anomaly detection (XAD) provides interpretable explanations for anomalies. Diverse stakeholders, including end-users, decision-makers, regulators, data scientists, and researchers, seek model explanations (Keshk et al., 2023; Sejr and Schneider-Kamp, 2021). These explanations can focus on the entire model, a specific domain subset, or individual predictions. Essentially, they explain the model's causal effects, not the domain's causal effects. The key value propositions of model explanations include enhanced trust, confidence, transferability, informativeness, causality, fair and ethical decision-making, model debugging, adjustment, and monitoring. These value propositions are particularly relevant given the increasing complexity of ICS architectures and the sophistication of cyberattacks targeting them.

**Figure 1:**
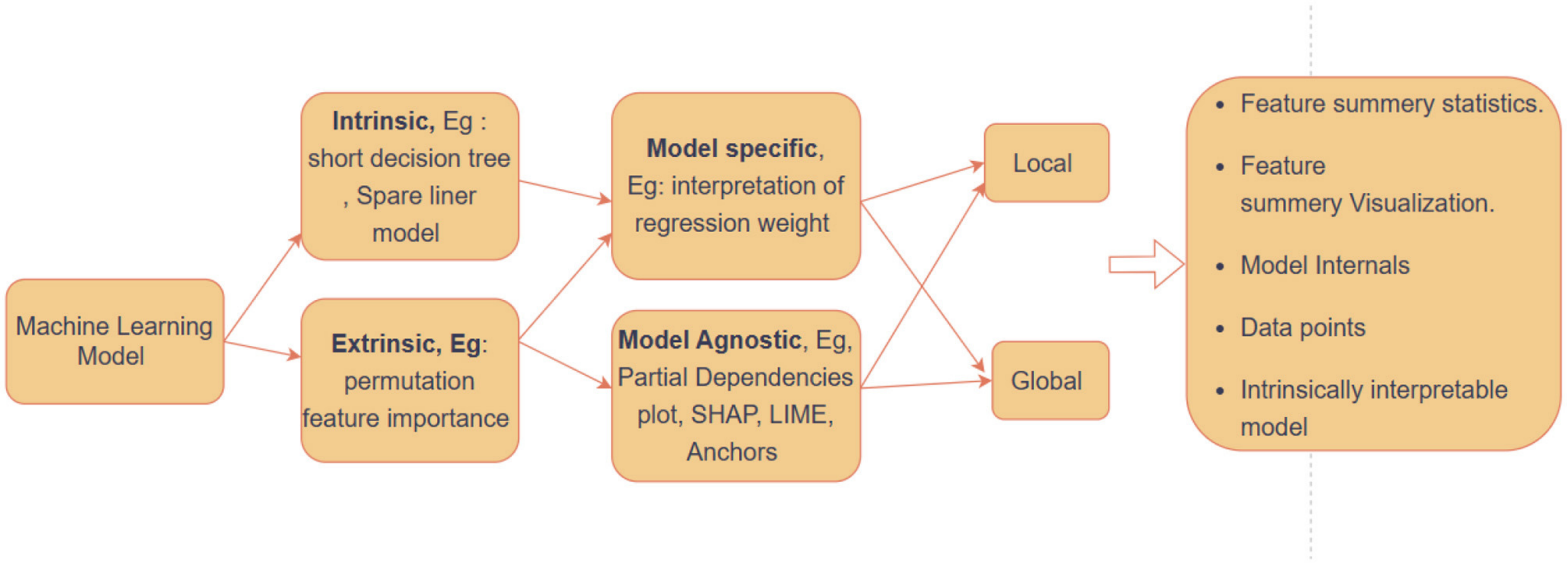
XAI concept and taxonomy (Wickramasinghe et al., 2021).

XAD uses statistical models, machine learning, and visualization, including feature analysis, Local Interpretable Model-agnostic Explanations (LIME), and Shapley values. XAD interpretation methods can be categorized as either global or local, depending on the scope of their model explanation (Wickramasinghe et al., 2021; Hwang and Lee, 2021), as shown in Figure 1. A global method provides explanations for all predictions made by the model, whereas a local method explains only one or a subset of the models predictions.

Interpretability varies across models. For example, Decision trees and k-nearest neighbor classifiers are considered interpretable due to their resemblance to human cognition (Sejr and Schneider-Kamp, 2021). Linear and logistic regression models are also interpretable due to their simplicity. Neural networks (Hwang and Lee, 2021), support vector machines, and ensemble trees are inherently uninterpretable, requiring *post hoc* explanation techniques (Sejr and Schneider-Kamp, 2021). While some methods are inherently interpretable, others require additional steps to explain their outputs. Sometimes, even after training a model, we might still want to understand how it makes decisions. There are two main ways to do this: by looking inside the model itself (model-specific), or by analyzing the data the model uses (model-agnostic). We have chosen a method called SHAP (SHapley Additive exPlanations) which is model-agnostic. This means it does not care about the inner workings of the model, but instead focuses on the data to explain why the model makes certain predictions.

In this study, the method we employed requires further explanation to fully comprehend the root cause of the anomaly. Since MGD are probabilistic models, they can be represented using mathematical formula. This facilitates understanding the model's inner workings and its predictive mechanisms. To understand how our model makes decisions, we use a technique called SHAP (SHapley Additive exPlanations). SHAP is like a tool that breaks down each prediction and tells us which features were most important in making that prediction (Hwang and Lee, 2021). SHAP is a widely used local explanation method that involves marginalizing Shapley values over every combination of feature existence in the point under explanation. However, a drawback of SHAP is its computational cost.

## 3 Methodology

The goal of the study is to investigate the effectiveness of correlation-based anomaly detection in identifying anomalies in ICS data. The proposed method's architecture is visually depicted in **Figure 3**, providing a clear and concise overview of the system's components and how they interact to achieve the method's functionality.

Since different operating conditions can affect features of industrial equipment in a time series, extracting correlations among features is crucial. To achieve this, we used sliding windows to make the correlations between features suitable for time series analysis. Sliding windows capture local patterns, adapt to data changes, and improve computational efficiency. Key parameters in a sliding window are samples, window size (length of each window), and step size (how the window slides). This approach generated subsequences of varying window sizes, where each window size represents the number of consecutive data points included in the analysis. For example, to create a sliding window of size W, the total number of samples N is divided by the timesteps T, which encompass all the features F involved in the observation, resulting in an input shape of (N, W, F). As the window size increases, the total number of samples decreases because the size of the window is subtracted from the total. To identify the optimal window size for analysis, we evaluate the reconstruction error of the LSTM-AE model across different window sizes. The reconstruction error, which measures the difference between the original input and the reconstructed output, indicates how well the model captured the underlying patterns and relationships in the data, hence the maximum root mean square error (RMSE) in reconstruction error. The LSTM-AE architecture involves creating data sequences with specified window sizes and shifts for training and testing. The model processes these sequences, using encoding layers to reduce dimensionality and decoding layers to reconstruct the original dimensions.

The proposed method leverages correlation-based anomaly detection (CAD) to uncover hidden relationships within datasets. The input is in the form of sequence of data records from segmented sliding windows, and the output consists of a report showing anomalies or not. First, the output of LSTM-AE is created as different windows and is then input to LCM in the form of a matrix. We utilized the notation of LCM with N parameters, represented as LCM = *r*_*i*_*j*_*N*_*N*. Here, the element *r*_*i*_*j* signifies the latent correlation between the i-th and j-th parameters. Pearson and Spearman correlations are used to construct the LCM for each window of LSTM-AE. The LCM captures the correlations between data points at different step sizes within a window. For example, examining a four-dimensional time series dataset within a window results in an LCM similar to the one provided in matrix M.


$$M=\left[\begin{array}{cccc} 1 & r_{12} & r_{13} & r_{14}\\ r_{21} & 1 & r_{23} & r_{24}\\ r_{31} & r_{32} & 1 & r_{34}\\ r_{41} & r_{42} & r_{43} & 1 \end{array}\right]$$

Once the LCM is calculated, it is squeezed into LCVs, denoted as *r*_*i*_ = {*r*_1_2, *r*_1_3, ..., *r*_*N*−1_*N*}. Latent correlation vectors (LCVs) were used to measure correlations between different monitoring data series within a specific time frame. Each extracted window corresponds to a specific LCV, resulting in multiple LCVs representing the equipments condition across various windows.

To model the latent correlations between features, we constructed a Latent Correlation Probability Model (LCMP) based on a MGD. This approach leverages the information obtained from applying a LCV analysis. The LCMP characterizes the probability distribution of the LCVs, allowing for the identification of anomalous LCVs and their relationship to the underlying model. The parameters of this Gaussian distribution were defined using the mean vector and covariance matrix derived from the correlation analysis. The mean vector represents the average value for each feature (variable) in LCV, while the covariance matrix captures the interdependencies between the individual features.

Equations 1, 2 define *r*_*i*_ as a vector containing the LCV values calculated for each window. Therefore, the data is segmented into multiple windows, *r*_*i*_ will hold a corresponding value for each window that reflects the strength of the local correlation within that window. Finally, we calculate the mean vector of the LCV data (average of all values in *r*_*i*_) to understand the overall level of local correlation across the entire dataset. The covariance, on the other hand, measures how the LCV values in different windows (represented by *r*_*i*_) tend to vary together.

**Figure 2:**
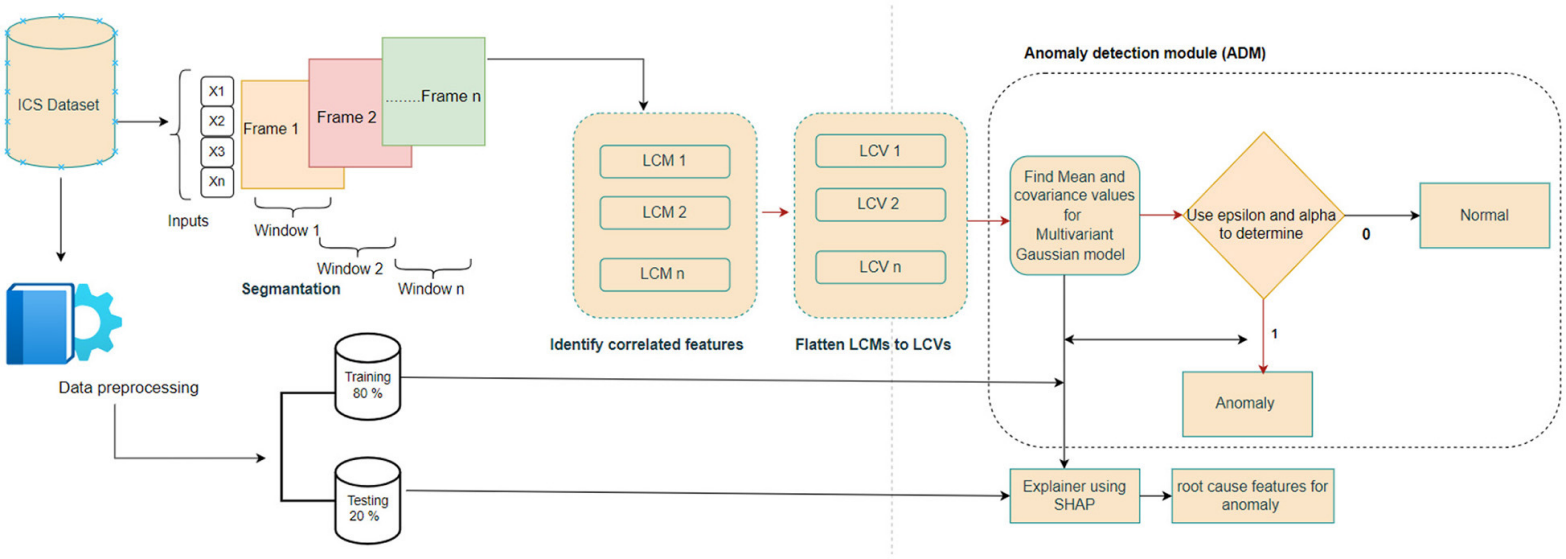
Proposed methodology architecture.

In Figure 2, the LCMP model, also referred to as the anomaly detection module (ADM), receives input from the LCV. ADM can be formulated for a given n-dimensional LCV {*r*_12_, *r*_13_, ..., *r*_*N*−1*N*_}, where r ϵ R, the mathematical expectation of all characteristics were calculated by the MGD and constructing a covariance matrix Σ for all characteristics. The model p(x) is then fitted by setting the Equations 2, 3.


(1) $$\begin{array}{l} \mu =\frac{1}{m}\overset{m}{\underset{i=1}{\sum }}r_{i} \end{array}$$

(2) $$\begin{array}{l} \Sigma =\frac{1}{m}\overset{m}{\underset{i=1}{\sum }}\left(r_{i}-\mu \right){\left(r_{i}-\mu \right)}^{T} \end{array}$$

Given a new example *x*, compute *p*(*x*) to flag an anomaly if *p*(*x*) < ϵ, where ϵ is the threshold.


(3) $$\begin{array}{l} p\left(x,\mu ,\Sigma \right)=\frac{1}{{\left(2\pi \right)}^{\frac{n}{2}}|\Sigma {|}^{\frac{1}{2}}}e\left(-\frac{1}{2}{\left(x-\mu \right)}^{T}\Sigma^{-1}\left(x-\mu \right)\right) \end{array}$$

The ADM searches for anomalous in the extracted windows containing correlation values and classifies each window as either normal or abnormal using a threshold-based method that uses the Alpha and Epsilon thresholds.

- **Using Alpha Threshold:** Each window LCV is processed as follows: (1) If all LCV correlation values are within the confidence bounds, the window is classified as normal (label 0). (2) Otherwise, the window is flagged as anomalous (label 1).
- **Using Epsilon Threshold:** it utilizes a pre-built MGD model to calculate the log probability density function of the extracted window data. It takes the window, the distribution model, and the best epsilon value as inputs to provide predictions for anomaly presence based on log probability distribution function (PDF) values and the epsilon threshold.

Algorithm 1 shows a procedure for generating data samples that follow and used by a MGD in the form of different window size. Two independent uniform random variables, *u*_1_ and *u*_2_, distributed uniformly on the interval [0, 1]. The random variable *z*_0_ is compute, following a standard normal distribution. This involves taking the square root of the negative of two times the natural logarithm of *u*_1_, and then multiplying this by the cosine of 2π*u*_2_. Hence, *z*_0_ is the output of this computation. Algorithm 2 shows a useful approach for analyzing the relationships between different features in a dataset by computing and sorting their Pearson correlation coefficients.

**Algorithm 1:** Generate the vector.

1: **Inputs:** *D*: ICS dataset, Mean_Vector: The mean vector, Covariance_matrix: The covariance matrix, Random numbers: u1 and u2, N_dimensions: dimensions of mean vector and covariance matrix
2: **Output:** Sample_Vector: A vector containing the sampled data points
3: Segment *D* into windows ←*D*
4: Compute the LCM ← windows
5: Flatten LCM (create LCV) ← LCM
6: Calculate LCMP ← LCV
7: Vector sample = []
8: **for** *i* from 1 to N_dimensions **do**
9: Generate *u*1 and *u*2 ← a uniform distribution in the range [0, 1]
10: Compute *z*_0_ ← $\sqrt{-2\ln\left(u1\right)}\times \cos\left(2\pi u2\right)$
11: Compute *z*_0_scaled__ ← $z_{0}\times \sqrt{\text{Covariance\_matrix}\left[i\right]\left[i\right]}$
12: Shifted value ← *z*_0_scaled__+Mean_Vector[*i*]
13: Append the scaled and shifted value to the samples vector
14: samples vector ←*z*_0_scaled__+ Shifted value
15: **end for**
16: **return** samples vector

**Algorithm 2:** Compute correlation coefficients.

1: **Input:** df_x_n_scaled: scaled features, key-value pair= k,v, Corr = Compute the Pearson correlation
2: **Output:** sorted_pearson: sorted list of Pearson correlation coefficients
3: Initialize empty correlation_coefficients = []
4: **for** each pair of col1, col2 in df_x_n_scaled **do**
5: Corr ← col1 and col2
6: pearson_dict ← |Corr|
7: **end for**
8: Initialize pearson_dict = []
9: **for** each k,v in pearson_dict **do**
10: **if** v is not null **then**
11: **return** pearson_dict ← k,v
12: **end if**
13: **end for**
14: Sort the k,v in pearson_dict in descending order
15: **return** sorted_pearson

To enhance interpretability, the proposed method employs XAD after development. SHAP assigns a Shapley value to each input feature, quantifying its contribution to the anomaly score. SHAP typically requires tabular data, which is obtained from the CAD model's output and test data, even though the model is unaware of this information. This is because SHAP is designed to interpret the output of machine learning models that utilize tabular data as input.

## 4 Experiments

### 4.1 Dataset description and exploration

In this study, we used the Secure Water Treatment (SWaT), HIL-based Augmented ICS Security, and IOT-Modbus datasets.

**Secure Water Treatment (SWaT):** it is a scaled-down version of an operational water purification plant. It consists of six stages, including raw water entry, chemical purification, ultrafiltration, inorganic substance removal, quality control, and tank water monitoring (Perales Gómez et al., 2020). The dataset contains 496,800 records from 28/12/2015 10:00:00 AM to 2/1/2016 2:59:59 PM, comprising sensors and actuators data, and an attack presence indicator.^1^ The 2015 A1 and A2 SWaT Dataset has 53 features, with 496,800 training records from normal operation and 449,919 records for attacks (Birihanu et al., 2022).

HIL- based augmented ICS security (HIL-HAI): it contains normal and attack data with 51 features for each instance.^2^ Each instance in the HIL-HAI 1.0 dataset represents a set of control loop operations used to monitor and adjust a process variable. The HIL simulator, turbine, water treatment system, and boiler are all included in the simulation. The dataset is divided into two training files and two testing files, each containing different attack scenarios and data recording intervals. It includes 63 features, encompassing both numerical and categorical data, and covers a 10-day period for training (normal data only) and 5.5 days for testing (including 38 attacks) (Shin et al., 2020).

**Internet of Things (IoT) Modbus:** these datasets represent a significant advancement in cybersecurity research, especially in the context of Industry 4.0. They serve as a crucial resource for evaluating the capabilities of AI-powered tools, with a particular focus on Machine Learning and Deep Learning algorithms. Spanning various data sources from both traditional IoT and Industrial IoT (IIoT) environments, these datasets offer valuable insights into emerging security challenges. One notable dataset within this collection is “IoT modus,” which comprises six (6) key features categorized into normal and attack scenarios.

The proposed model combined the normal and attack records for each dataset, resulting in 946,719 records for the SWaT dataset, 995,404 records for the HAI dataset, and 287,156 records for the IoT Modbus dataset. All features were retained to account for potential anomalies across any attribute. Categorical features were encoded using one-hot encoding, and numerical features were normalized using min-max scaling to ensure consistency in the data preprocessing steps.

### 4.2 Experiment setup

This experiment aims to evaluate the effectiveness and generalizability of the proposed method compared to baseline methods. Initially, experiments were conducted to determine the optimal window size for the proposed MGD model, which effectively captures latent correlations in the data. Different window sizes were tested to detect the optimal one, ranging from 2 to 512. The test set was generated with window sizes W_n_ = {2^3^, 2^4^, 2^5^, 2^6^, 2^7^, 2^8^, 2^9^} = {8, 16, 32, 64, 128, 256, 512}. The primary motivation for comparing performance using a window-based approach is that the original data was segmented into windows using LSTM-AE methods. This segmentation is necessary because the complete working process of an ICS, from start to finish, collects a vast amount of data points, including information from edge devices (sensors and actuators). Different working conditions can affect the time series parameters of these edge devices. The window-based approach was chosen due to limited computational resources, the need to handle temporal dependencies, background knowledge about the datasets, and the goal of enhancing model performance. Additionally, segmenting data into windows for LSTM-AEs allows for efficient learning, better generalization, and effective anomaly detection or pattern recognition in time-series tasks.

We utilized an LSTM-AE to identify the optimal window size, experimenting with various sizes and selecting 8 as the best choice based on the lowest reconstruction error and early stopping to avoid overfitting. Instead of using default hyperparameter values, we employed grid search to select the best hyperparameters, including 5 hidden layers, Adam optimizer, mean squared error loss, Sigmoid activation, 20 epochs, and a batch size of 128.

**Figure 3:**
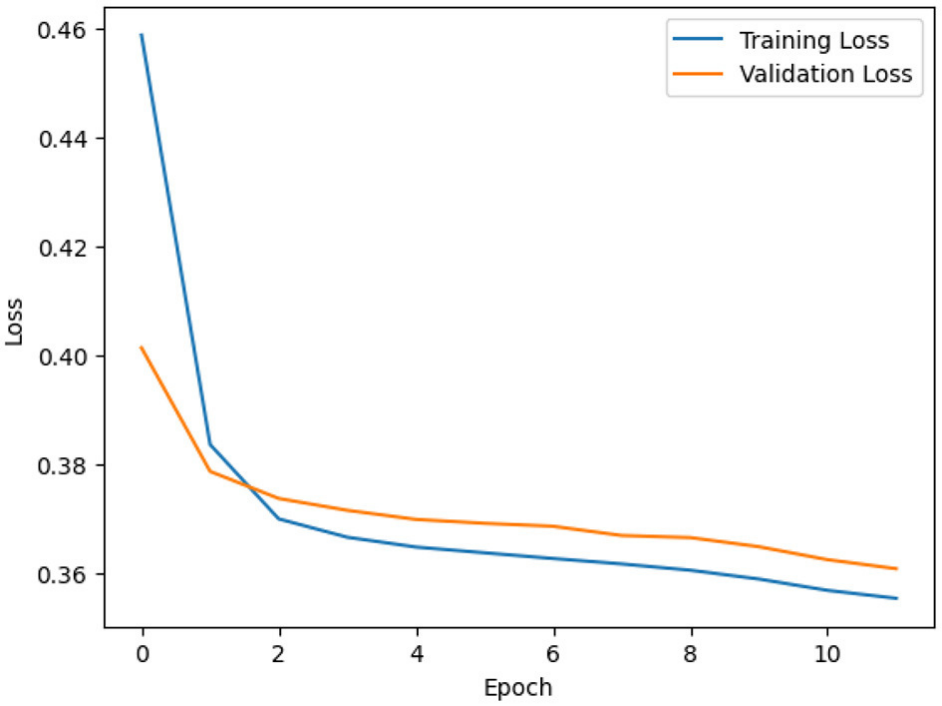
LSTM-AE approach training and validation loss using SWaT training data.

The training and validation losses of the proposed LSTM-AE methods decreased as the number of epochs increased, as depicted in Figure 3. Both training and validation losses showed no overfitting problem, indicating the effectiveness of regularization and dropout techniques in preventing overfitting. Following LSTM-AE training for automatic window size detection, we applied the sliding window technique to compute the LCM. This approach divides the dataset into fixed-size windows, enabling the capture of temporal dynamics and variations within the data. Additionally, the dataset was preprocessed, including dropping duplicate records, scaling with min-max scalar, applying one-hot encoding for categorical features, and splitting into 80/20 training and testing data. This experiment was conducted using Colab Pro, the TensorFlow framework, and the Keras library, all part of the Python 3.10.12 environment, on hardware with two Intel(R) Xeon(R) CPUs (2.20 GHz) and 50.99 GB RAM. Our code is publicly available at a GitHub repository.^3^

### 4.3 Experiment 1

The SWaT dataset presented a significant challenge due to its large size. This dataset contained a massive volume of data points (495,000 samples) with a high number of features (53). To effectively analyze these characteristics, we utilized non-overlapping sliding windows for data segmentation.

We began the experiment with a window size of 8, encompassing all 53 feature instances within each window. This configuration aimed to capture localized data changes while maintaining the complete feature set for analysis in the large dataset. Subsequently, we systematically increased the window size across different experiments. This exploration allowed us to assess the trade-off between capturing sufficient historical context and computational efficiency for processing such a vast amount of data.

The proposed method achieved highest performance on the SWaT dataset, surpassing 96.0% precision, indicating a very low rate of false positives. This high precision translates to a remarkable F1-score exceeding 84.0%, demonstrating a well-balanced performance between precision and recall. Interestingly, these optimal results were achieved with a window size of 128 data points, deviating from the consistent 256 window size observed in other datasets. This highlights the potential influence of dataset size on optimal window selection for the proposed method.

### 4.4 Experiment 2

The HIL-HAI dataset presented a unique challenge due to its complex relationships among features, requiring thorough analysis and modeling techniques to derive valuable insights. This dataset contained a total of 309,601 samples, each with a significant number of features (54). To effectively analyze these complex relationships, we again employed non-overlapping sliding windows for data segmentation.

Similar to the IOT-Modbus dataset, we initiated the experiment with a window size of 8, encompassing 35 feature instances. This approach aimed to balance capturing localized changes while incorporating some historical context within the high-dimensional data. We then systematically increased the window size across multiple experiments. This exploration allowed us to assess the model's performance in identifying anomalies while considering the impact of window size on computational efficiency for such a large dataset.

The proposed method achieved better results on the HIL-HAI dataset. It surpassed 96.0% precision, indicating a low rate of false positives. While recall remained moderate, exceeding 58.0%, the overall F1-score remained significant at over 0.53%. Notably, these optimal performance metrics were obtained, once again, using a window size of 256 data points. This consistency across datasets strengthens the argument for the effectiveness of this window size in capturing relevant temporal information for anomaly detection in ICS data.

### 4.5 Experiment 3

The experiment utilized the IOT-Modbus dataset, containing 222,856 samples with 6 features each. To segment the data for time-series analysis, we employed non-overlapping sliding windows. We explored various window sizes to determine the optimal configuration for anomaly detection performance. Our initial exploration started with a window size of 8, encompassing 4 feature instances within each window. This configuration aimed to capture localized data changes while maintaining some historical context. We subsequently increased the window size incrementally across multiple experiments. This exploration allowed us to assess the impact of window size on the model's ability to identify anomalies within the ICS data.

The proposed method achieved promising results on the IOT-Modbus dataset, exceeding 52.0% precision, 58.0% recall, and a significant F1-score of 0.53%. The performance degradation observed in the IOT-Modbus dataset compared to others may be attributed to its smaller size and the differences in data collection methods, which were conducted using simulation test beds. The limited sample size might hinder the model's ability to generalize, while simulation data may lack the full complexity and variability of real-world scenarios, potentially leading to discrepancies in model performance. Further investigations and refinements in data collection strategies, along with the potential augmentation of the dataset with additional samples, could help mitigate these challenges and enhance the performance of the proposed mechanism on the IOT-Modbus dataset. Notably, these optimal performance metrics were obtained when employing a window size of 256 data points. This finding highlights the importance of selecting an appropriate window size for capturing both relevant temporal information and avoiding overfitting on specific data patterns. Table 1 provides a comprehensive breakdown of the performance metrics achieved with different window sizes for the selected ICS datasets. This analysis offers valuable insights into the interplay between window size and model performance.

**Table 1:** Experimental results summary.

**Data set**	**Window size**	**Precision**	**Recall**	**F1-score**	**AUC**
SWat 2015	8	0.54	0.50	0.11	0.50
	16	0.51	0.50	0.12	0.50
	32	0.64	0.68	0.65	0.69
	64	0.93	0.78	0.83	0.77
	**128**	**0.96**	**0.78**	**0.84**	**0.78**
	256	0.96	0.77	0.83	0.76
	512	0.94	0.75	0.81	0.75
HIL-HAI 20.7	8	0.53	0.57	0.53	0.56
	16	0.53	0.59	0.54	0.60
	32	0.54	0.57	0.55	0.58
	64	0.57	0.60	0.58	0.60
	128	0.61	0.64	0.62	0.63
	**256**	**0.96**	**0.77**	**0.87**	0.58
	512	0.79	0.72	0.75	**0.76**
IoT_Modbus	8	49	49	0.04	0.48
	16	0.50	0.48	0.19	0.48
	32	0.50	0.47	0.24	0.46
	64	0.50	0.49	0.24	0.49
	128	0.50	0.54	0.24	0.51
	**256**	**0.52**	**0.58**	**0.53**	**0.57**
	512	0.51	0.53	0.07	0.53

## 5 Result and discussion

**Figure 4:**
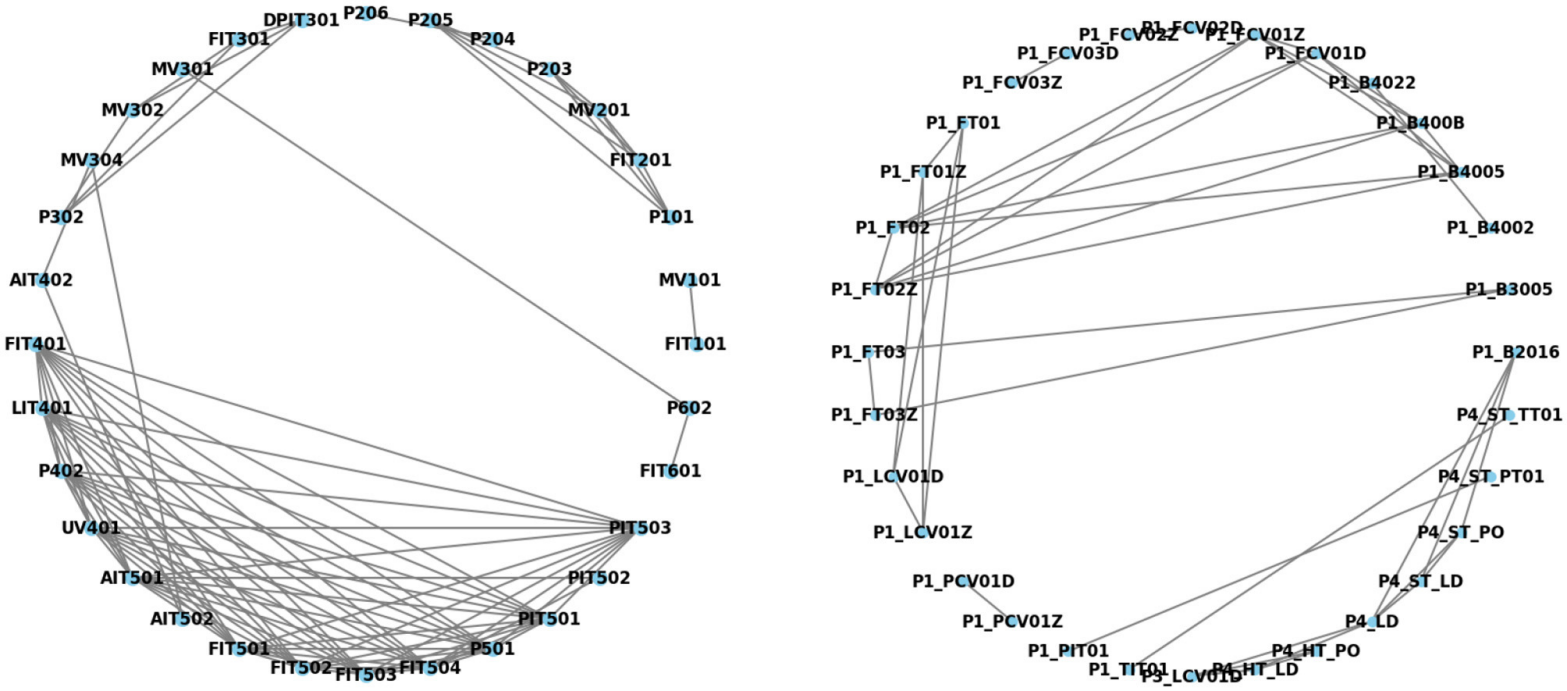
Correlation map for SWaT **(left)** and HIL- HAI **(right)**.

The investigation of correlation analysis techniques within ICS showed its effectiveness for identifying interconnected devices. The basic assumption here is that closely linked features, as identified by techniques such as the Pearson correlation coefficient, indicate devices that influence one another. The coefficient itself detects statistically significant relationships between features, implying a proportional change in one might induce a change in the other. The results of correlation analysis are typically visualized using correlation matrices or maps to provide a valuable tool for understanding the interdependence within features of an ICS dataset. As shown in Figure 4, the correlation map was used to find the high-priority features based on strong correlations across each dataset. For instance, for SWaT dataset feature PIT503, FIT401, PIT501, FIT504, and FIT503 are in strong correlation with other features. In addition, for HIL-HAI dataset features P1_FT02 have high correlation with P1_FCV01Z, P1_ FCV01D, P1_B400B, and P1_B4005. Hence any change in these features may impact the entire network and are critical to be monitored.

This study investigated extracting latent correlations from windowed datasets. We achieved this by utilizing the upper triangle of the correlation matrix (excluding redundant diagonal and lower portions) and flattening it into a vector called Latent Correlation Vectors (LCVs). This captured the essential correlation information within each window. We then collected LCVs from all windows, representing the extracted latent correlations for the entire dataset. The latent correlation model was built using a MGD. Its mean vector and covariance matrix were estimated. To identify anomalies, we implemented two thresholds: alpha (α) and epsilon (ϵ). Our experiments demonstrated that alpha is more effective. This is because, for confidence intervals in MGDs, alpha directly reflects the confidence level, providing a clear understanding of data uncertainty. While epsilon might be suitable in specific cases, alpha offers a more robust and interpretable framework. The resulting anomaly score is represented by either a single LCV or a number of LCVs, with 0 indicating normal and 1 signifying an anomaly. Table 1 presents the detailed experimental results. The investigation focused on the performance of the proposed method across different window sizes. Window sizes 128, 256, and 512 outperformed others in terms of precision, recall, and F1-score for SWaT2015, HIL-HAI 20.7, and IoT Modbus datasets, respectively. To evaluate the performance of the proposed method we also used the Area Under the Curve (AUC) metric. The higher AUC score indicates better performance, which ranges from 0 to 1^4^. Interestingly, the experiments revealed that the optimal window size for achieving the best AUC varied depending on the dataset. While a window size of 128 performed best in the first experiment, a window size of 256 yielded the best results in the second and third experiments. These findings imply that the characteristics of a dataset, such as the number of samples and the dimensionality of its features, significantly impact the optimal window size required to achieve a maximized AUC score. Since the AUC and recall in Table 1 are very similar; this indicates that the model performs consistently well in identifying positive instances across different thresholds.

Besides, the performance of the proposed methods is influenced by the dataset size, which is determined by the chosen window size. However, the variation in window size has been widely discussed as a key factor in how data size and time span affect anomaly detection model results. A large window size may include information from multiple activities, increasing the computational load and decreasing the model's reactivity. Conversely, a small window size might split some activities into multiple consecutive windows, triggering anomaly detection too frequently without achieving high accuracy. While decreasing the window size allows for faster anomaly detection, a very small window size can be prone to classification errors.

**Figure 5:**
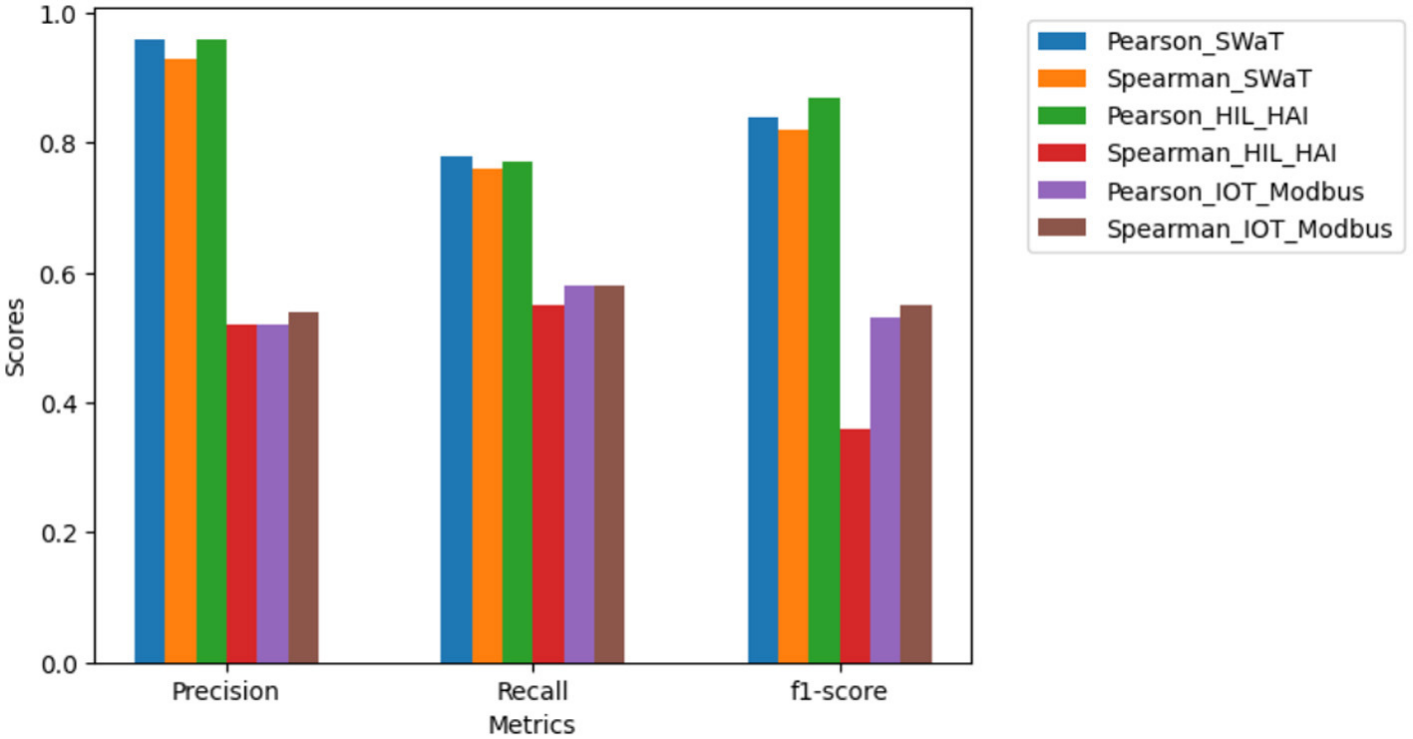
Comparison of correlation methods across datasets.

This experiment investigated the impact of Spearman correlations. The window size for each dataset was chosen based on the one achieving the maximum performance. The results show that Pearson correlation is more effective in anomaly detection for the proposed method, as it captures linear relationships and underlying patterns better, while Spearman correlation, which deals with monotonic relationships, is less effective. Thus, Pearson correlation is the preferred choice to determine the threshold points for the proposed method. A threshold of 0.7 was applied to select only features exhibiting the strongest positive correlations. Since the correlation matrix is symmetrical, only the upper triangle was considered for analysis. Figure 5 shows the comparison of correlation methods for each data set at their respective maximum window sizes.

**Figure 6:**
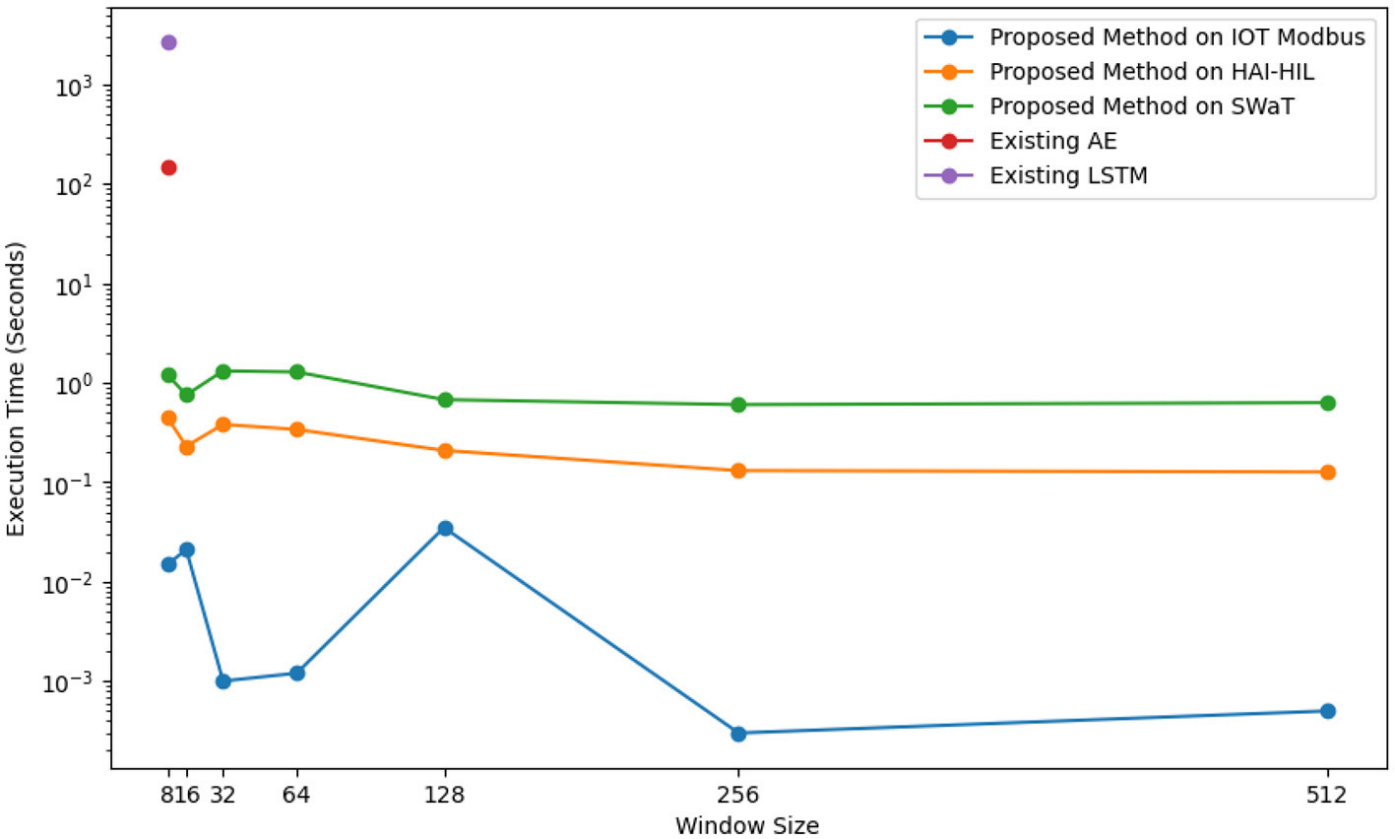
Execution time comparison: proposed vs. existing methods on datasets.

**Figure 7:**
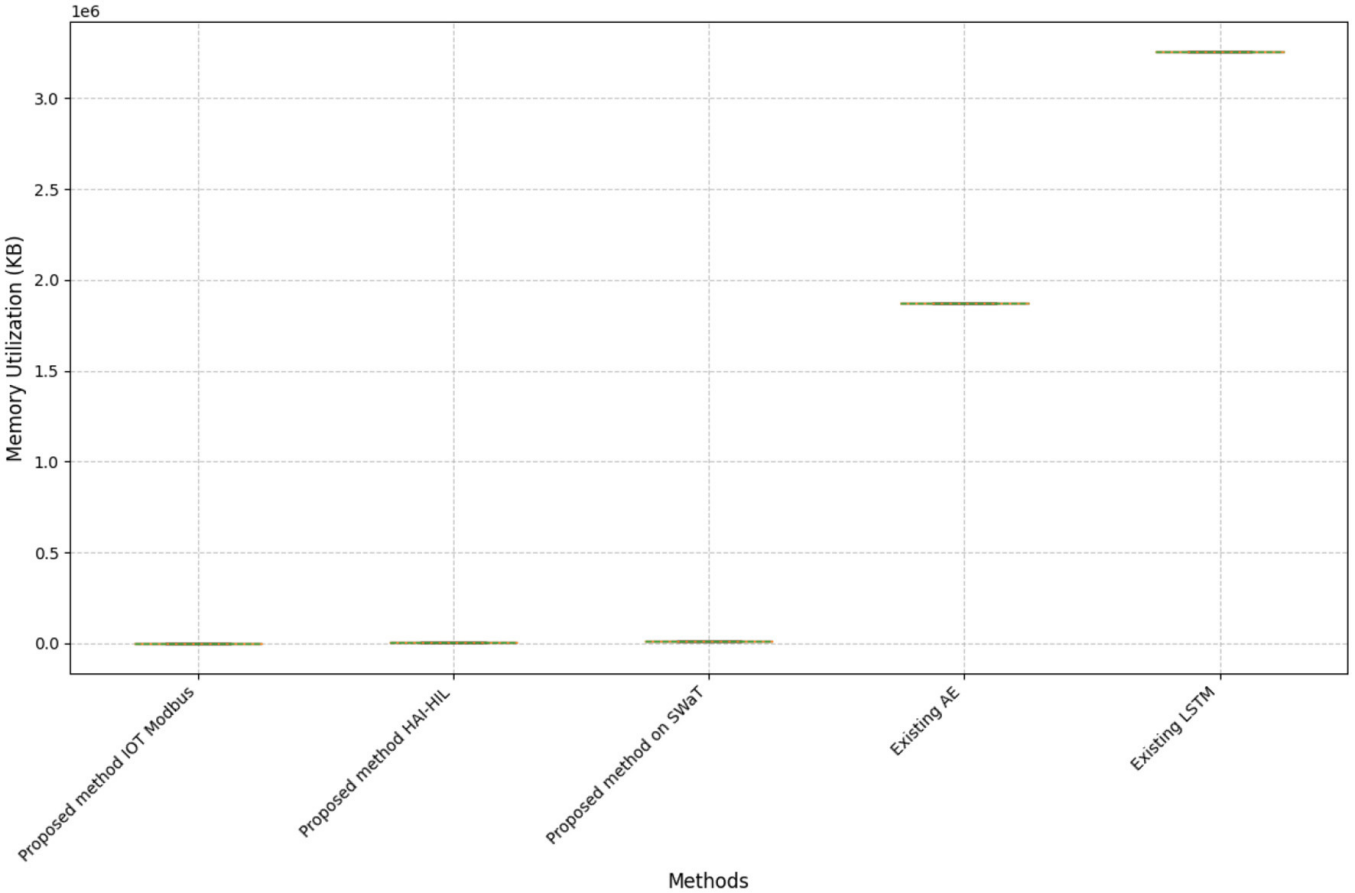
Memory utilization comparison: proposed vs. existing methods on datasets.

To evaluate the resource efficiency of our proposed method, we measured both execution time and memory utilization. Figures 6, 7 compares execution times for existing and proposed methods across different datasets. The goal of utilizing memory and time analysis in the proposed method is to ensure efficient resource allocation. This is particularly critical for small ICS devices, such as sensors and actuators, deployed in ICS applications, as these devices often operate under constrained computational resources and limited memory capacity. Figure 6 presents a single point, depicting the results of both existing AE and LSTM methods in terms of the execution time. Interestingly, the proposed method exhibits a trend where execution time decreases as the window size increases. This observation suggests that the model's proficiency in reducing redundant computations at larger window sizes might contribute to more efficient processing. This improvement is because processing smaller, manageable segments reduces computational overhead and optimizes resource usage, allowing for more efficient training and testing.

The experiment demonstrated that the proposed method significantly outperforms the existing system in terms of both execution time and memory usage.This is because the proposed method focuses only on training specific and informative segments of the data. These segments were identified using windows generated by the LSTM-AE, which effectively helped to discover patterns within the data. In contrast, traditional autoencoder (AE) and LSTM models process the entire dataset at once, leading to increased processing time and memory requirements. This highlights the efficiency and effectiveness of our proposed method.

Furthermore, the comparison results with previous researchers shown that the proposed method perform well in terms of precision and F1 scores, indicating its better ability to accurately classify anomalies. Although Autoencoder (AE) and Long Short-Term Memory (LSTM) models had lower precision, they demonstrated slightly better recall compared to the proposed model. As shown in Table 2, the proposed method recall value is lower than that of state-of-the-art models. A low recall value indicates that the model is missing positive cases. This could be due to the imbalanced nature of our dataset or the fact that we did not select the appropriate features. Several characteristics of the ICS dataset make feature selection challenging for anomaly detection methods. Firstly, ICS data is often complex because many sensors and actuators produce a lot of data. It makes hard to select the most important features due to the sheer volume and complexity of the data. Secondly, ICS data can be sparse, meaning there might be missing information or data collected at irregular times. This makes it tough to find useful features because some data points might be missing. Also, the working principle of ICS environments are always changing. This means an important feature at one time might not be important later on. Lastly, IoT data often shows complicated relationships between sensors or actuators and the system they are monitoring (Sung et al., 2022). According to these challenges, the proposed method focuses on developing techniques that can identify anomalies in ICS without requiring feature selection. In addition, our proposed method, based on the Multivariate Gaussian Distribution, shows lower recall and F1 scores than One-Class SVM (OC-SVM) and Isolation Forest (IF) due to its reliance on Gaussian assumptions. In contrast, OC-SVM and IF are more flexible and robust, effectively handling diverse data patterns.

**Table 2:** Comparison of the proposed and existing methods.

**Model**	**Precision**	**Recall**	**F1-score**
LSTM (Perales Gómez et al., 2020)	0.81	0.87	0.82
OCSVM (Bernieri et al., 2019)	0.84	0.90	0.93
IF (Sung et al., 2022)	0.86	0.81	0.95
OCSVM (Inoue et al., 2017)	0.92	0.69	0.79
AE (Bernieri et al., 2019)	0.79	0.85	0.81
**Proposed method**	**0.96**	0.78	**0.84**

Thus, the finding of the correlation between features in the proposed method offers a viable alternative to commonly used deep learning approaches like LSTM and AE, delivering comparable performance with a potentially different precision-recall trade-off. Table 2 indicates the comparison of the proposed method and existing work with precision, recall, and F1-score. This study explored the potential of leveraging all features within ICS data. By analyzing their correlations through method using Pearson correlation (Figure 5), we can create a comprehensive picture of normal system behavior. This flags the way for building scalable anomaly detection models that identify deviations from established relationships between features, potentially leading to earlier and more accurate threat detection within ICS environments. Additionally, using XAD with the correlated model via the SHAP method allows us to interpret the model's inner workings, understand anomalies directly, and identify their root causes within the context of ICS. The root cause of anomalies within ICS datasets utilizes XAD techniques, such as SHAP. SHAP incorporates two key functionalities: feature importance analysis, which highlights the most influential variables contributing to the anomaly, and causal inference methods, which uncover causal relationships between variables and anomalies.

While the results of the proposed methods indicate that correlation-based anomaly detection for ICS achieves good performance, our aim here is to investigate whether the proposed method can significantly assist domain engineers in efficiently identifying and verifying abnormal behavior in sensors and actuators. To achieve this, we employ SHAP to uncover how individual features contribute to the anomalies the model predicts. SHAP provides powerful explanations for the predictions of any machine learning model, including our proposed method. In the case of our MGD model, SHAP can explain the contributions of each feature and their interactions to the final prediction. Interpreting the proposed method decisions and prioritizing system checks and maintenance, respectively, could enhance operational efficiency, saving operators valuable time in the process.

**Figure 8:**
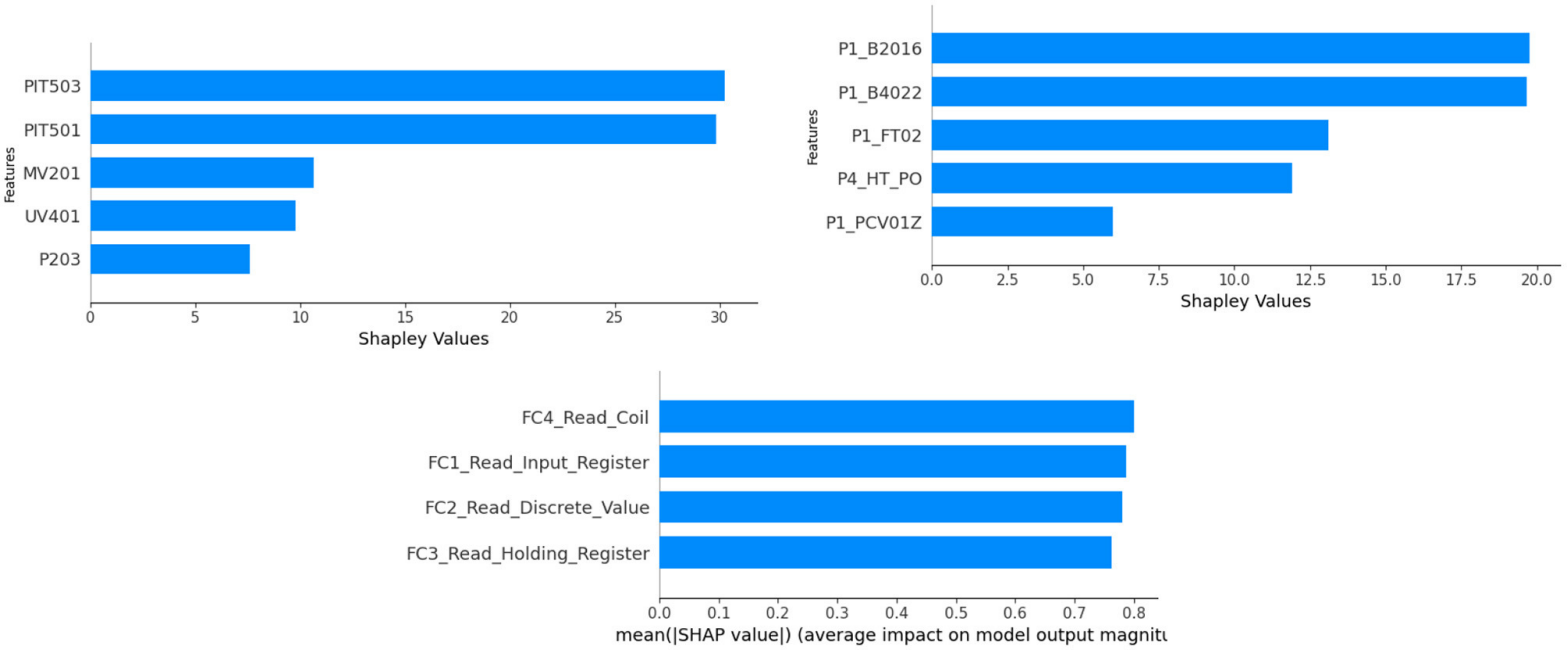
The root cause features for SWaT, HAI-HIL, and IOT_modbus ranked from highest to lowest.

Figure 8 illustrates the distribution of SHAP values across all computed data points, highlighting the top five most important features based on their impact. This result suggests that we could identify the root causes of anomalies. We chose the highest performance score window for each dataset, as determined by the MGD method. The x-axis represents SHAP values, while the y-axis displays features ordered from most to least important. Features are arranged in ascending order of importance for each dataset. However, in this study, we were unable to further validate the suggestions for the proposed model from the end users and domain engineers directly involved in testbed development.

## 6 Conclusion and future work

In today's data-driven world, businesses use anomaly detection to find hidden irregularities in vast datasets. Correlation-based anomaly detection is gaining popularity for its precision in analyzing variable associations.

This study aimed to apply a technique to identify anomalies in multivariate datasets by analyzing correlation patterns and identifying the root cause of anomalies using XAD. Our method detects collective anomalies within specific timeframes, as point anomalies may not exhibit significant correlation variations across multiple variables. To analyze the effect of window size on anomaly detection, we employed non-overlapping sliding windows of various sizes. Our findings highlight the critical role of selecting an appropriate window size and using LSTM-AE algorithms for segmenting the ICS datasets. These steps are fundamental for achieving accurate final anomaly predictions. Following data segmentation, we implemented an anomaly detection module that utilizes a MGD model with different window sizes. This module effectively predicts anomalies within the system by employing a threshold mechanism based on epsilon and alpha values. Moreover, it goes beyond simply identifying anomalies by effectively pinpointing their root cause within the ICS domain. However, training machine learning models on large datasets with sliding windows requires careful resource optimization (execution time and memory). This ensures efficient deployment on resource-constrained devices like small ICS units.

While the proposed method prioritizes local explainability, it can be extended to incorporate other techniques, such as global and *post-hoc* explainability, for a more comprehensive understanding. This analysis, designed for offline applications, operates on batch data to identify features responsible for anomalies in the ICS testbed. Future research could involve using real-time data streams from real-world scenarios to assess the significance of root cause identification in practical settings and to evaluate the robustness and generalization ability of our proposed model in a streaming data environment. Additionally, we plan to incorporate a variety of effective deep learning anomaly detection algorithms, including TimesNet.

## Data Availability

Publicly available datasets were analyzed in this study. This data can be found at: https://itrust.sutd.edu.sg/itrust-labs_datasets/dataset_info/ and https://www.usenix.org/conference/cset20/presentation/shin.

## Footnotes

^1^ https://itrust.sutd.edu.sg/itrust-labs-home/itrust-labs_swat/

^2^ https://www.usenix.org/conference/cset20/presentation/shin

^3^ https://github.com/Ermiyas21/Explainable-correlation-AD

^4^ https://yassineelkhal.medium.com/confusion-matrix-auc-and-roc-curve-and-gini-clearly-explained-221788618eb2
